# Minoxidil weakens newly synthesized collagen in fibrotic synoviocytes from osteoarthritis patients

**DOI:** 10.1186/s40634-023-00650-8

**Published:** 2023-08-21

**Authors:** Stefan Sarkovich, Peter P. Issa, Andrew Longanecker, Davis Martin, Kaitlyn Redondo, Patrick McTernan, Jennifer Simkin, Luis Marrero

**Affiliations:** 1 Department of Orthopaedic Surgery, Louisiana State University Health Sciences Center, 2021 Perdido St., Center for Advanced Learning and Simulation, 7th floor, New Orleans, LA 70112 USA; 2 School of Medicine, Louisiana State University Health Sciences Center, 2020 Gravier St., Lions Building, 5th floor, New Orleans, LA 70112 USA; 3 Morphology and Imaging Core, Louisiana State University Health Sciences Center, 533 Bolivar St., Clinical Sciences Research Building, 5th floor, New Orleans, LA 70112 USA; 4 Department of Physiology, Louisiana State University Health Sciences Center, 533 Bolivar St., Clinical Sciences Research Building, 4th floor, New Orleans, LA 70112 USA

**Keywords:** Osteoarthritis, Synovium, Synovial fibroblasts, Fibrosis, Collagen type I, Pyridinoline, Lysyl hydroxylase, Minoxidil, Arthroplasty, Knee

## Abstract

**Purpose:**

Synovial fibrosis (SFb) formation and turnover attributable to knee osteoarthritis (KOA) can impart painful stiffness and persist following arthroplasty. To supplement joint conditioning aimed at maximizing peri-operative function, we evaluated the antifibrotic effect of Minoxidil (MXD) on formation of pyridinoline (Pyd) cross-links catalyzed by *Plod2*-encoded lysyl hydroxylase (LH)2b that strengthen newly synthesized type-I collagen (COL1) in fibroblastic synovial cells (FSCs) from KOA patients. MXD was predicted to decrease Pyd without significant alterations to *Col1a1* transcription by FSCs stimulated with transforming growth factor (TGF)β1.

**Methods:**

Synovium from 10 KOA patients grouped by SFb severity was preserved for picrosirius and LH2b histology or culture. Protein and RNA were purified from fibrotic FSCs after 8 days with or without 0.5 µM MXD and/or 4 ng/mL of TGFβ1. COL1 and Pyd protein concentrations from ELISA and expression of *Col1a1*, *Acta2*, and *Plod2* genes by qPCR were compared by parametric tests with α = 0.05.

**Results:**

Histological LH2b expression corresponded to SFb severity. MXD attenuated COL1 output in KOA FSCs but only in the absence of TGFβ1 and consistently decreased Pyd under all conditions with significant downregulation of *Plod2* but minimal alterations to *Col1a1* and *Acta2* transcripts.

**Conclusions:**

MXD is an attractive candidate for local antifibrotic pharmacotherapy for SFb by compromising the integrity of newly formed fibrous deposits by FSCs during KOA and following arthroplasty. Targeted antifibrotic supplementation could improve physical therapy and arthroscopic lysis strategies aimed at breaking down joint scarring. However, the effect of MXD on other joint-specific TGFβ1-mediated processes or non-fibrotic components requires further investigation.

## Introduction

Synovial fibrosis (SFb) is a hallmark of knee osteoarthritis (KOA) characterized by aberrant type 1 collagen (COL1) deposition in the synovial subintima that constricts the joint capsule to effectively impart painful stiffness and limitations in active range of motion (ROM) [17]. Although the definitive treatment for KOA is total knee arthroplasty (TKA), severe arthrofibrosis in the synovium and other peri-articular soft tissues can persist post-operatively [1], potentially hindering patients from maximizing functional gains. Since pre-TKA ROM, which is significantly associated with the severity of SFb [17], is the strongest predictor of post-operative ROM [37], refining strategies to condition articular soft tissues peri-operatively will likely improve surgical outcomes and lower the risk for arthrofibrosis complication.

KOA stiffness prior to TKA can be associated with synovitis and/or SFb at various grades and stages of severity [17]. Post-TKA stiffness is reported by up to 30% of patients [40], accounts for 28% readmission [38], causes 10% of revisions within five years of surgery [39], and drives a 20% rate of patient dissatisfaction with TKA outcomes [15]. Strategies for dissociating peri-operative arthrofibrosis such as manipulation under anesthesia (MUA) and arthroscopic lysis of adhesions are expensive and not guaranteed for long-term relief of stiffness related to the ongoing turnover of fibrous COL1 deposits [9, 40]. COL1 processing involves complex post-translational modifications on assembly, including conversion of lysine to hydroxylysine by lysyl hydroxylase (LH)1, 2a, 2b, and 3 [46]. Particularly, overexpression of LH2b encoded by the procollagen-lysine,2-oxoglutarate 5-dioxygenase (*Plod*)*2* gene corresponds to increases in pyridinoline (Pyd) cross-links, leading to over-hydroxylation of telopeptide lysine residues [46]. Pyd increases the tensile strength of COL1 fibrils and their resiliency to degradation by proteases [45]. This phenomenon has been consistently confirmed to occur during the engineering of collagenous networks by cancer-associated fibroblasts in metastatic cancer and various fibroproliferative processes, including pannus formation and SFb in a mouse model of KOA [34] and in the diseased synovium of KOA patients [35].

As a vasodilator and potassium channel activator, Minoxidil (MXD) was approved by the United States Food and Drug Administration (FDA) to treat hypertension and alopecia [51] but has been repurposed as an antifibrotic in various studies for its negative effect on the formation of hydroxylysine and hydroxyallysine cross-links [26]. Moreover, MXD has been suggested to have an inhibitory effect on the canonical transforming growth factor (TGF)β1 pathway in lung myofibroblasts after treatment in a mouse model of bleomycin-induced-pulmonary fibrosis that resulted in weakened bronchioalveolar COL1 fibrillogenesis [41]. However, studies on the *Plod2*-mediated formation of COL1 networks that promote migration of metastatic neoplasms suggest that MXD may directly interfere with transcription of *Plod2* [12] and that inhibition of the *Plod2*-LH axis has an adverse effect on the structural integrity and assembly of mature COL1 rather than COL1 transcription and synthesis itself [19]. Similarly, application of MXD to fibroblasts derived from pediatric patients with talipes equinovarus (i.e., clubfoot) in vitro caused alterations in COL1 processing without cytotoxicity [21]. Underpinned by this paradigm, our study was designed to evaluate the antifibrotic efficacy of MXD on primary fibroblastic synovial cells (FSCs) isolated from the synovium of KOA patients classified with low to severe SFb in the presence or absence of TGFβ1 levels typically measured in KOA synovial fluid [44, 50].

## Methods

### Patients and sample collection

This level III study was approved by the Institutional Review Board (#986) of the Louisiana State University Health Sciences Center in New Orleans and all procedures were executed in agreement with relevant guidelines after informed consent from 350 patients. Participants were adults > 18 years old with clinical and radiographic evidence of end-stage KOA and eligible for TKA. TKA on all 350 patients was performed by one arthroplasty surgeon using equivalent surgical technique, implants, and rapid recovery protocol. Samples were collected from discarded suprapatellar synovium from routine anterior compartment synovectomy during TKA. Synovial tissues were washed in saline, bisected, and half preserved in 10% zinc-buffered formalin. The remainder was submerged in basal synoviocyte media (Cell Applications, San Diego, CA) containing 10% fetal bovine serum and 10% dimethyl sulfoxide for controlled, slow freezing from room temperature to -80 °C for 24 h in a Mr. Frosty™ (ThermoFisher, Waltham, MA) container followed by storage in a liquid nitrogen cryounit at vapor phase. Based on calculated histological SFb severity values, 350 KOA patients were grouped into quartiles, and five patients randomly selected from the top (*n* = 5; highest SFb cohort) and bottom quartiles (*n* = 5; lowest SFb cohort) for this study. However, each group was required to include three women and two men around the median age of 67 and body mass index (BMI) around 34.5, calculated from the entire patient population consented to bank synovium in the facility.

### Synovium histology and quantitative immunohistochemistry

Formalin-fixed synovial tissues were paraffin processed, sectioned at 5 µm onto slides, dried at 60 °C for 15 min, deparaffinized with xylene and graded ethanols, from absolute to 80%, rehydrated into distilled water, and stained by picrosirius (PS) technique to classify patients by histological SFb severity based on published methods [17] for a total of 5 patients with high (> 54% PS-stained collagen area) and 5 patients with low (< 40% PS-stained collagen area) SFb with corresponding synovial tissues cryopreserved for cell isolation. To measure LH2b expression, serial paraffin sections from those stained by PS of all 10 patients were mounted on slides, including an additional two for negative controls, dried at 60˚C for 45 min, deparaffinized, submerged in pH 6.0 citrate buffer (Abcam, Cambridge, UK) at 60 °C for 17 h, and allowed to cool at room temperature (RT) for 20 min. Slides were washed in phosphate-buffered saline (PBS) where indicated. Following treatment with Protein Block (Abcam), sections were incubated overnight at 4 °C with a primary antibody against LH2b (Abcam; rabbit polyclonal; 2 µg/mL) and washed. Notably, monoclonal antibodies specific to LH2b are unavailable for immunolabeling of formalin-fixed, paraffin-embedded sections of human tissue. Negative control slides were incubated in antibody diluent only. Slides were then incubated with a rabbit F(ab’)^2^ secondary antibody (Jackson Immunoresearch, West Grove, PA, US; goat polyclonal; 4 µg/mL) conjugated to Alexa 594 for 45 min, washed, and coverslipped with Prolong Diamond (Thermo Fisher). Two fields per sample containing synovial intima and subintima were captured at 200 × magnification using a FV1000 laser scanning confocal microscope (Olympus of America, Center Valley, PA) at 200x equipped with a 592 nm laser diode and relevant photodetectors. The emitted fluorescence from the PS dye or LH2b immunolabel was segmented from the photomicrographs with a watershed algorithm using Slidebook software (3i, Denver, CO) and specific signal pixel areas automatically measured, divided over a constant, total pixel area calculated for the entire field of view, and values averaged between fields [17, 24].

### Isolation and culture of patient FSCs

Synovial tissues cryopreserved in freezing media were rapidly thawed at 37˚C, minced, and incubated in Roswell Park Memorial Institute (RMPI) media supplemented with type IV collagenase at 0.5 mg/mL for 90 min at 37˚C in a shaking water bath set to 180 revolutions per minute (rpm). The cell suspension was filtered through a 70 μm mesh and centrifuged at 250xg for 10 min. The cell pellet was resuspended in synoviocyte growth medium (SGM; Cell Applications) supplemented with 10% FBS, 1% streptomycin, 1% penicillin, 0.1% gentamycin, and 0.1% amphotericin. After counting by hemacytometer, each patient set of FSCs were seeded into multiple T-25 flasks containing SGM at a density of 7 × 10^5^ cells and expanded through one passage in a sterile environment at 37 °C and 5% CO_2_ for flow cytometry and experimental treatments.

### Immunophenotyping FSCs by flow cytometry

After trypsinization with Trypsin–EDTA (Cell Applications) for 30 s, 250,000 cells were washed with PBS and immunolabeled for one hour with working concentrations of: CD45-APC (BioLegend, San Diego, CA; #368512; 1:20), CD31-APC (BioLegend; #303103; 1:20), CD90-BV421 (Becton Dickinson (BD), Franklin Lakes, NJ; #562556; 1:20), CD73-Cy7 (BD; #561258; 1:20), CD105-BB700 (BD; #566528; 1:20), CD55-PE (BD; #561901; 1:10) and the Fixable Viability Dye efluor™ 780 (ThermoFisher, Waltham, MA; #65–0865-14; 1:100), washed, fixed in 1% methanol-free formaldehyde, and analyzed in a FACS Canto (BD). Cells were gated by forward/side scatter for size, granularity, singlet subtyping, and viability. All live cells were evaluated for immunolabeling using unstained cells as negative controls and a commercial human fibroblast-like synoviocyte (HFLS; Cell Applications) line as a positive control.

### Stimulation and treatment of cultured FSCs

Carrier-free, recombinant TGFβ1 (R&D Systems; #7754-BH-005/CF) was dissolved in 10 mM citric acid to a 50,000 ng/ml stock for dilution in SGM to 1, 2, or 4 ng/mL working solution [2]. MXD (Sigma-Aldrich; #M4145) was dissolved in 96% ethanol to a 119 mM stock for dilution to 0.5 mM in SGM [21]. To test the effect of exogenous TGFβ1 to stimulate COL1 output, myofibroblast differentiation, and proliferation, HFLS were seeded at 7,000 cells/cm^2^ onto each well of 8-well glass chamber slides in SGM to include duplicates of non-treated (NT) or treated HFLS with TGFβ1 diluted at 1, 2, or 4 ng/mL for 48 h. At endpoint, all cells were briefly washed with pre-warmed PBS, fixed in 10% zinc formalin, washed, acrylic wells removed, and the slides permeabilized in acetone pre-chilled to -20 °C for 5 min and washed. One set of slides was co-immunolabeled with rabbit anti-COL1 (Abcam; ab21286) and mouse (clone 1A4) anti-SMA (Agilent, Santa Clara, CA; M0851) primary antibodies diluted at 1 and 4 µg/mL in antibody diluent (Abcam), respectively, and co-incubated for 1.5 h at RT and washed. An additional set was incubated with a mouse monoclonal antibody against Ki67 (clone SP6; Abcam; ab16667) at 5 μg/mL. For indirect co-labeling of COL1 and SMA or single labeling of Ki67, secondary F(ab’)^2^ antibodies against rabbit and mouse IgG conjugated to Alexa 488 and Alexa 594 (Jackson ImmunoResearch, Philadelphia, PA), were co-applied at 2 μg/mL each, or the latter applied individually with 4',6-Diamidino-2-Phenylindole, Dihydrochloride (DAPI; ThermoFisher) at 300 nM for nuclear and Ki67 co-labeling, and incubated for 45 min at RT. All slides were washed and coverslipped with Prolong Diamond (ThermoFisher). Two, 200x photomicrographs per duplicate well were captured using a multi-Argon laser and 592 nm diode of the FV1000 confocal with corresponding photodetectors. Percent changes in COL1 and SMA immunolabeled protein content were analyzed relative to total cellular area using Slidebook mask thresholding and morphometry functions [24]. Proliferation percentages were calculated from manual counts of Ki67-positive nuclei over total nuclei labeled with DAPI [25].

An equivalent 7,000 cells/cm^2^ of HFLS or FSCs from each of the 10 patients were seeded in 24-well plates in SGM. After 24 h, cells were incubated in SGM supplemented with or without 4 ng/mL of TGFβ1 and/or 0.5 nM MXD and repeated every 48 h for 8 days. Experimental groups with three replicates per sample type and/or condition included HFLS or FSCs from each patient in SGM 1) non-treated (NT) controls and those supplemented with either 2) TGFβ1 only, 3) MXD only, or 4) TGFβ plus MXD.

### COL1 and Pyd output measures

At experimental endpoint, HFLS and KOA FSCs were trypsinized on ice, washed, and dissociated using an ice-cold nonionic buffer from the phenol-free Ambion™ PARIS™ kit (ThermoFisher; #AM1921) for co-extraction of RNA and protein following manufacturer instructions. TURBO DNAse (ThermoFisher; #AM2238) was integrated to abrogate DNA contamination. RNA quality and concentration were determined using a 2100 bioanalyzer (Agilent). To determine and normalize protein concentrations for the enzyme linked immunosorbent assays (ELISA), we employed a bicinchoninic acid assay (Abcam; #ab102536) and an xMark™ microplate reader (Bio-Rad, Hercules, CA). COL1 and Pyd output was measured by sandwich (Abcam; #ab210966) and competitive (Novus, Centennial, CO; #NBP2-82,518) ELISAs, respectively, per manufacturer guidelines and validated standards with optical densities measured at 450 nm with the microplate reader.

### Quantitative polymerase chain reaction (qPCR)

A custom qPCR array with validated PrimePCR™ primers (Bio-Rad) for human *COL1α1* (qHsaCED0002181), *Plod2* (qHsaCED0045587), and *Acta2* (qHsaCID0013300) was used on RNA extracts from all experimental cell lines and normalized against housekeeping mitochondrial ribosomal protein L13 (MRPL13; qHsaCED0046878) [28, 48]. The array is integrated with an RNA quality assay and technical controls for the reaction (synthetic DNA), reverse transcription (synthetic RNA transcript), and contamination with genomic DNA. Complimentary (c) DNA was synthesized using 100 ng of RNA from each treatment group with a Superscript™ IV First-Strand Synthesis Kit (ThermoFisher; #18,091,050) and a PTC-200 thermocycler (MJ Research, Saint Bruno, Quebec) per manufacturer guidelines. The cDNA was diluted 1:10 in SYBR® Green Supermix (Bio-Rad; #1,708,880) and nuclease-free water. Commercially available, total RNA from control HFLS (Cell Applications) or RNA isolated from experimental NT control HFLS were used as sample calibrators for qPCR analyses of relative expression in experiments involving HFLS or patient FSCs, respectively. A Lightcycler 480 (Roche, Indianapolis, IN) was used to assay the array and cycle threshold (Ct) values processed using the ΔΔCt method, analyzed, and log-scaled.

### Statistical analysis

Prism 9 (GraphPad, San Diego, CA) was used to analyze all results with α = 0.05. Student’s t-test was used to compare IHC values and Pearson’s correlation (R) employed to associate histological metrics with output measures in vitro. One-way analysis of variance (ANOVA) with Tukey adjustment for multiple comparisons was used to analyze ELISA output and differences in ΔΔCt values with Tukey or Sidak for multiple comparisons.

## Results

### Patient SFb severity status correlates to fibrous output from cultured FSCs

The 10 patients included in this study were 60% women, with median (interquartile range or IQR) of 66.5 (59.5–73.0) years old and BMI of 34.28 (29.72–40.42) kg/m^2^. No statistically significant differences were measured between patients’ median (IQR) when grouped into high versus low SFb cohorts for age (67.0 (51.0 – 71.0) versus 66.0 (55.0 – 73.0)) or BMI (36.4 (29.72 – 39.06) versus 32.15 (30.04 – 40.4)).

**Fig. 1:**
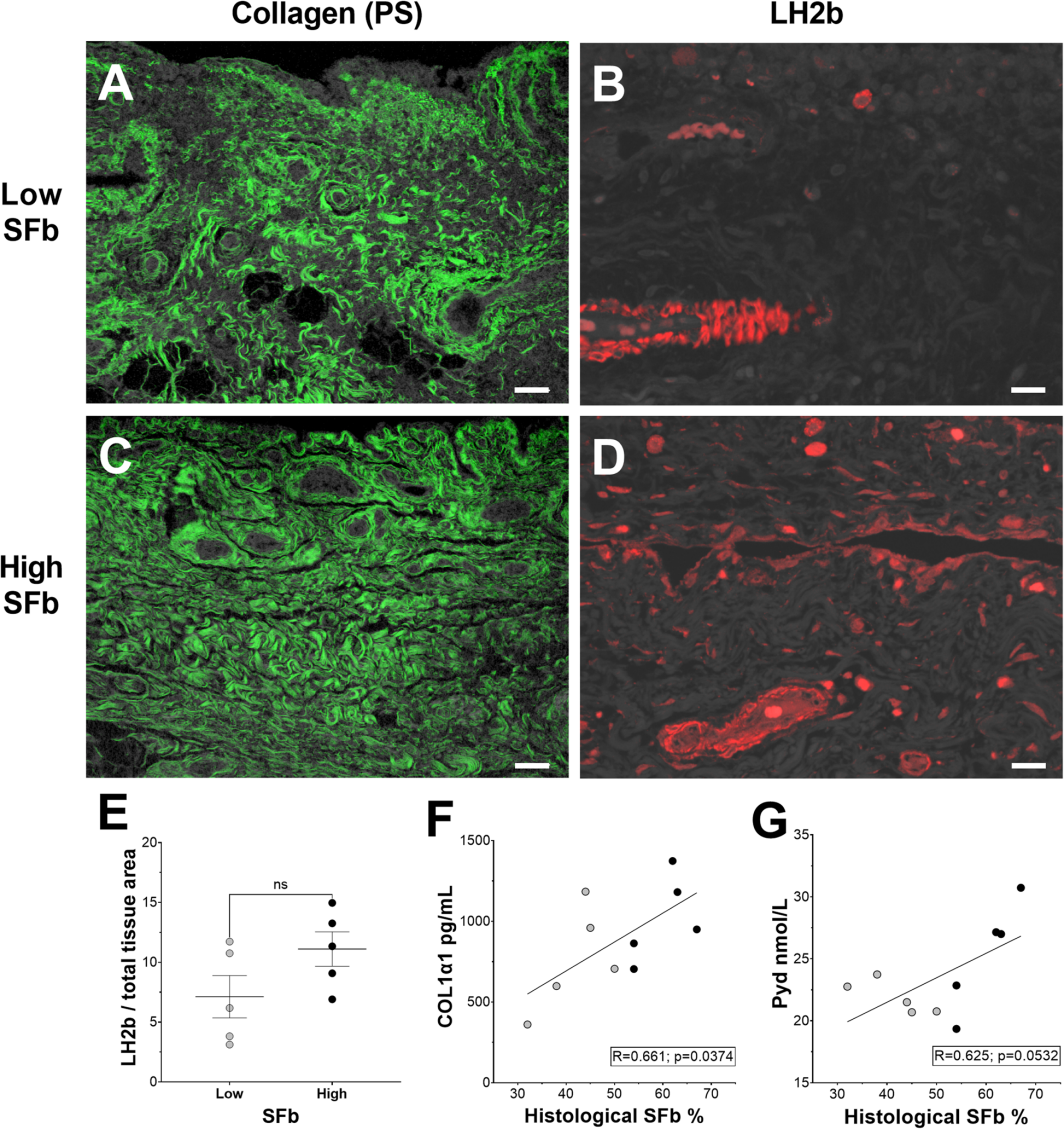
(**A, C**) The severity of fibrous collagen deposition, or SFb, (green) measured from confocal photomicrographs of PS-stained, paraffin sections from the synovium of KOA patients is relative to (**B**, **D**) LH2b (red) immunolabeled content (**E**) measured as pixel area of LH2b signal over total tissue area of serial sections from those stained with PS. Scale bars = 100 µm; ns = not significant (**F**, **G**) COL1 and Pyd output measured by ELISA from homogenates of FSCs grouped by low (gray circles) or high (black circles) SFb, represented by gray or black circles, associate to histological SFb severity values

Histological SFb measurements from PS-stained sections were used to group 10 KOA patients into high (> 50% COL1) or low SFb cohorts (Fig. 1A, C). Consecutive sections from those stained with PS were immunolabeled for LH2b (Fig. 1B, D). Although the mean ± standard error of the means (SEM) LH2b expression measured from the high SFb group (11.11 ± 1.44) was 43.6% higher than that of the low SFb group (7.13 ± 1.76), the difference observed was not significant (*p* = 0.118; Fig. 1E).

Flow cytometry on the isolated FSCs from all 10 patients showed a mean ± SEM 99.5 ± 4.34% of cells were reactive to one or more cluster differentiation (CD) markers typically found on the surface of FSCs: CD90, CD73, CD105, or CD55. A mean ± SEM 60.58 ± 16.32% of cells were positive for all four CD markers. Additionally, 99.9 ± 0.02% of FSCs were unreactive to antibodies against hematopoietic CD45 and endothelial CD31. COL1 values from protein fractions of untreated patient FSCs measured by ELISA displayed a moderately high correlation (*R* = 0.661; *p* = 0.0374) relative to histological SFb severity measures. A moderate association was calculated between Pyd output measured from the same protein fractions and histological SFb (*R* = 0.625; *p* = 0.0532) (Fig. 1F and G).

### MXD decreases fibrogenic output from KOA FSCs regardless of SFb status

**Fig. 2:**
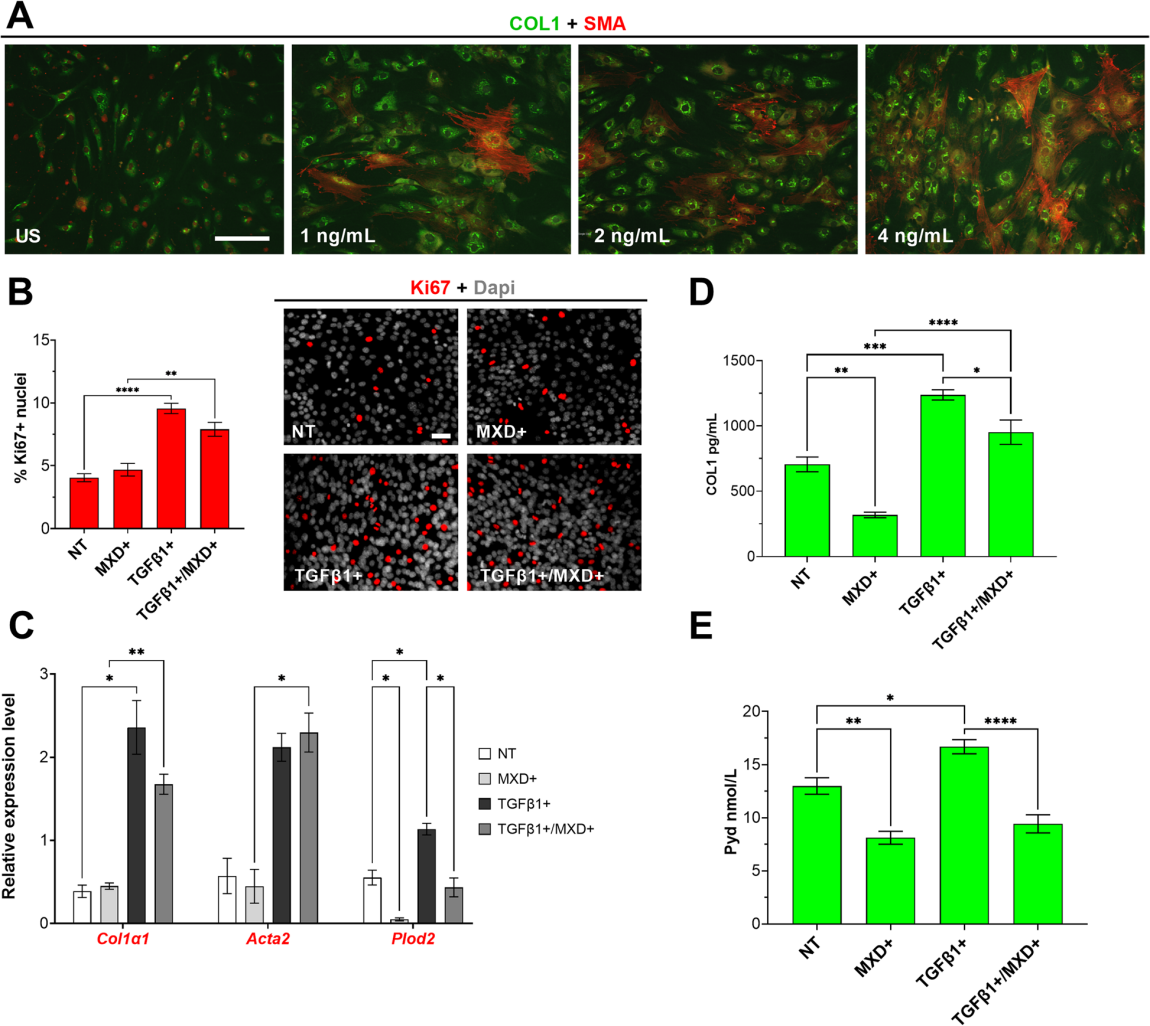
**A** Immunocytochemical detection of intracellular COL1 production (green) and SMA-positive stress fibers (red) increase in a dose-dependent manner 48 h after inoculation of unstimulated (US) HFLS with recombinant TGFβ1. Bar = 25 μm (**B**) Changes in proliferation rate between samples that remained non-treated (NT) and those stimulated with TGFβ1 and treated with MXD individually or in combination was measured by immunocytochemistry of Ki67-positive (red) nuclei over total DAPI (gray)-stained nuclei. Bar = 25 μm (**C**) Expression of target genes measured by qPCR from RNA extracted from HFLS treated under various conditions for 8-days was analyzed by ΔΔCt method and log scaled following statistics. **D**, **E** COL1 and Pyd output were measured by sandwich and competitive ELISA, respectively, from homogenized HFLS in the presence and absence of MXD and/or TGFβ1. *p ≤ 0.05, **p ≤ 0.01, ***p ≤ 0.001, ****p ≤ 0.0001, no asterisk: *p* > 0.05

**Fig. 3:**
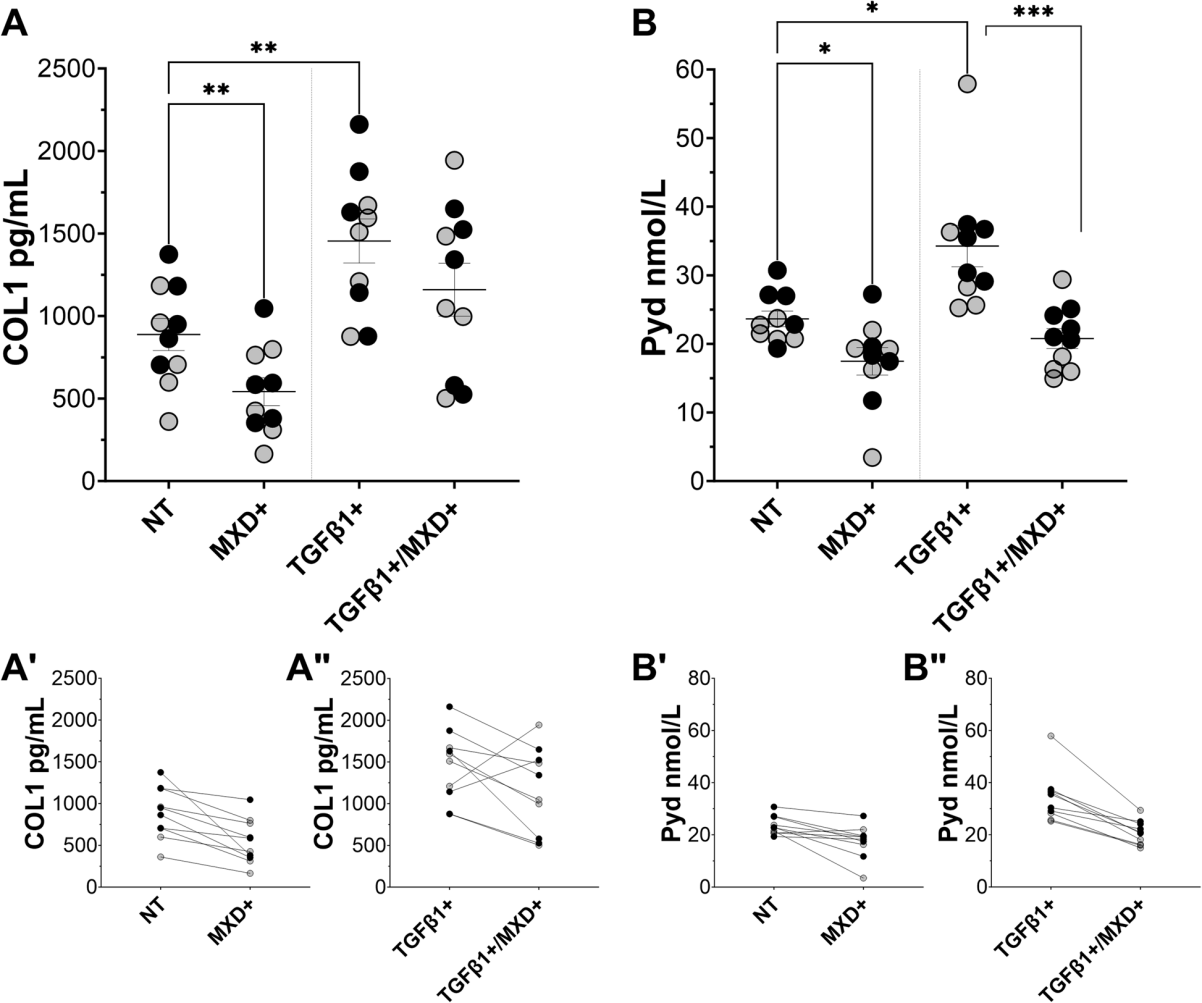
FSCs from patients grouped by high (black circles) and low (gray circles) SFb remained untreated (NT) or were treated with MXD in the presence or absence of TGFβ1 stimulation. **A**-**A**” COL1 output was measured and compared between groups and the effect of MXD treatment on unstimulated or TGFβ1-stimulated FSCs from each patient is shown. **B**-**B**” The effect on Pyd content was also measured under the same conditions. *p ≤ 0.05, **p ≤ 0.01, ***p ≤ 0.001, no asterisk: *p* > 0.05

In unstimulated (US) conditions (i.e., no TGFβ1; Fig. 2A), MXD treatment of HFLS decreased intracellular COL1 by 42.73% (*p* = 0.0073) from NT controls (Fig. 2D). On average, COL1 output was also decreased in unstimulated KOA patient FSCs (38.96%; *p* = 0.0249) after MXD treatment compared to NT, regardless of KOA patient SFb severity status (Fig. 3A). However, MXD+ FSCs from the low SFb group showed a highly significant decrease in COL1 production (*p* = 0.006) compared to NT. An equivalent comparison between NT and MXD+ FSCs from the high SFb group showed a weaker response (*p* = 0.057) (Fig. 3A’). In brief, the most robust and reasonably significant effect of MXD on COL1 processing was observed in low COL1-producing cell lines: naïve HFLS and KOA patient FSCs in the absence of pro-fibrotic stimulus and FSCs from the low SFb cohort.

In addition to lower COL1 output, a mean 46.21% and 23.13% decrease in Pyd was measured from unstimulated, MXD-treated HFLS (*p* = 0.0025) and KOA patient FSCs (*p* = 0.019), respectively, relative to corresponding NT controls (Figs. 2E and 3B). However, when Pyd values in the absence or presence of MXD were compared between FSCs grouped by low or high SFb status, MXD showed a more significant and consistent decrease in Pyd in FSCs from KOA patients classified with high SFb (*p* = 0.025) than those with low SFb (*p* = 0.155) (Fig. 3B’). These SFb severity-dependent differences persisted when comparing Pyd corresponding to FSCs from NT low (*p* = 0.0112) versus high SFb (*p* = 0.007) groups stimulated with TGFβ1 (Fig. 3B”).

### MXD consistently alters Plod2 transcription and Pyd formation in KOA FSCs under TGFβ1 stimulation

**Fig. 4:**
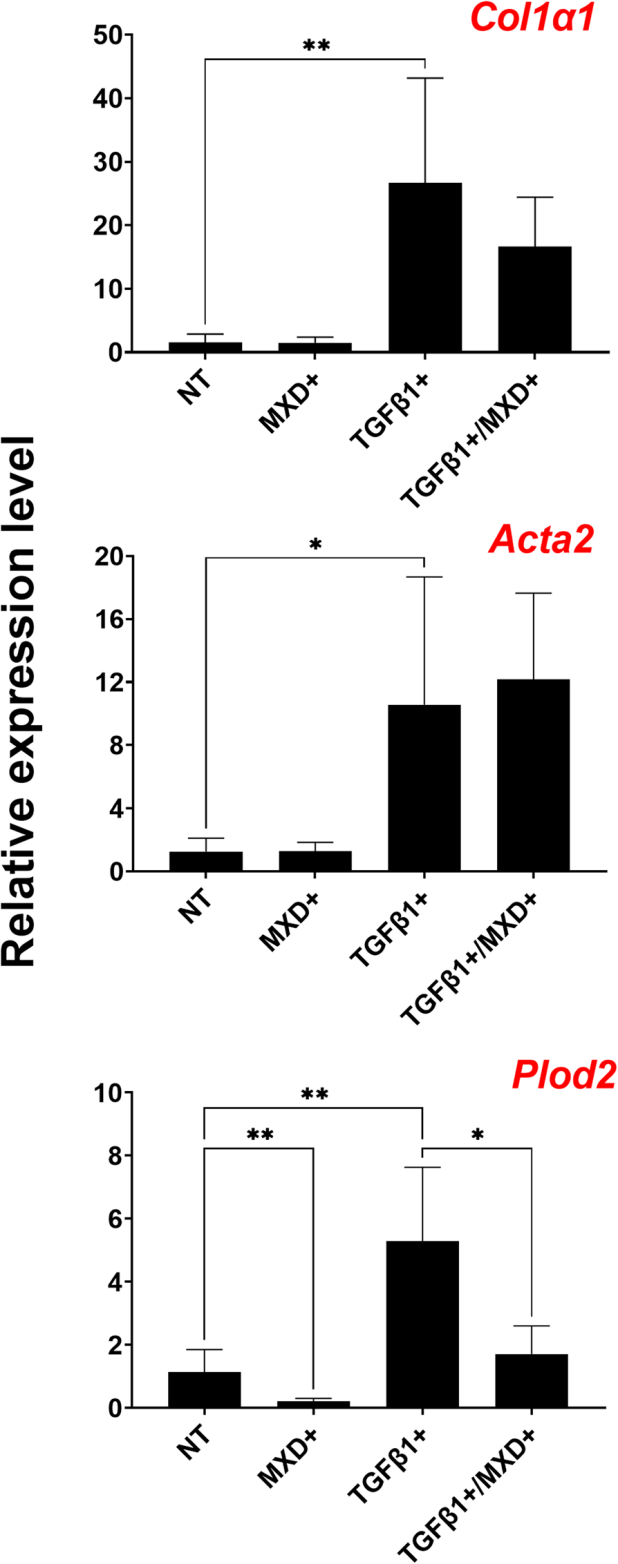
Comparison of ΔΔCt ratios to evaluate changes in expression of select genes fundamental to COL1 synthesis, myofibroblast transition, and LH2b expression for processing of Pyd cross-links in KOA patient FSCs under the different experimental conditions. Data represent mean ± SEM for *n* = 10 KOA patient primary FSCs run in duplicate. *p ≤ 0.05, **p ≤ 0.01, no asterisk: *p* > 0.05

Since KOA patients generally present with elevated concentrations of TGFβ1 in synovial fluid and peri-articular tissues [44], exogenous application of recombinant TGFβ1 was integrated to the experimental treatment with MXD on naïve HFLS and KOA patient FSCs. First, the fibrogenic effect of exogenous TGFβ1 application was evaluated on naïve HFLS prior to testing on KOA patient FSCs. Groups of HFLS seeded at equivalent density per well with increasing doses of TGFβ1 elevated COL1 expression and the incidence of SMA-positive FSCs in a dose-dependent manner (Fig. 2A), with the most robust distribution observed in HFLS stimulated with 4 ng/mL of TGFβ1. Accordingly, and in tandem with KOA patient FSCs, experimental groups of HFLS were treated with MXD only, TGFβ1 only, or in combination for 8 days. Proliferation significantly increased after stimulation with exogenous TGFβ1 with (*p* = 0.0015) or without (*p* =  < 0.0001) MXD administration (Fig. 2B). A mean ± SEM baseline of 705.50 ± 56.60 pg/mL of COL1 was measured from protein extracts of NT HFLS after 8-day incubation. Compared to baseline and relative to total protein concentration calculated from samples in each treatment group, stimulation of naïve and MXD-treated HFLS with TGFβ1 increased COL1 by 75.48% (*p* = 0.0002) and 199.31% (*p* < 0.0001), respectively, validated by corresponding elevated expression of *Col1a1* (*p* = 0.0354 for NT vs. TGFβ1+ and *p* = 0.0034 for MXD+ vs. TGFβ1+ /MXD+); Fig. 2C). The mean increase in COL1 from NT baseline that was measured from TGFβ1-stimulated HFLS was reduced by 23.09% when MXD was co-administered with TGFβ1 (*p* = 0.0224; Fig. 2D). Despite these results, the addition of MXD to TGFβ1-stimulated cultures did not appear to significantly alter TGFβ1-mediated transcription of *Col1a1* for COL1 synthesis and *Acta2* for myofibroblast differentiation (Fig. 2C). Like HFLS, cultures of KOA FSCs in the presence or absence of TGFβ1 stimulation but treated with MXD displayed significant downregulation of *Plod2* (*p* = 0.0179 for MXD+ only and *p* = 0.0446 for MXD+ / TGFβ1+ ; Fig. 4), in agreement with decreasing Pyd concentrations in corresponding treatment groups (*p* = 0.0179 for MXD+ only and *p* = 0.0446 for MXD+ / TGFβ1+ (Fig. 3B).

The mean COL1 baseline for all 10 NT KOA patient FSCs was 59.91% higher than that for NT HFLS but mean ± SEM differences between NT FSCs from low (761.9 ± 142.8) versus high SFb (1015.0 ± 246.6) were not significant (*p* = 0.2102). Regardless of SFb status, all KOA patient FSCs were responsive to TGFβ1 stimulation, measured by increased fibrillogenesis (i.e., COL1 plus Pyd formation). Overall, the mean concentration of COL1 in TGFβ1-stimulated FSCs by endpoint was 63.78% higher (*p* = 0.0011) than NT. In combination with TGFβ1 stimulus, MXD decreased mean COL1 in FSCs from 1455.0 ± 133.4 pg/mL in NT groups to 1159.0 ± 161.1 in corresponding MXD+ groups but this difference was insignificant (Fig. 3A), even when comparing TGFβ1+ alone versus MXD+ plus TGFβ1+ FSCs in low SFb (*p* = 0.497) or high SFb (*p* = 0.148) (Fig. 3A”). Interestingly, 2/10 cell lines under TGFβ1 stimulation did not respond to MXD independent of histological SFb classification (Fig. 3A”).

Compared to NT groups, a significant increase in Pyd was observed on both HFLS (*p* = 0.0172) and patient FSCs (*p* = 0.0158) after exposure to exogenous TGFβ1 (Figs. 2E and 3B). However, MXD had a significant effect on the amount of Pyd cross-links on both control HFLS and KOA FSCs despite TGFβ1 stimulation with a mean 43.54% in lysates from MXD+ HFLS (*p* < 0.0001) and 39.24% decrease in lysates from KOA FSCs (*p* = 0.0004) in combination with TGFβ1 compared to HFLS or KOA FSCs stimulated with TGFβ1 only. Notably, the effect of MXD on Pyd was greater when TGFβ1 was present, and FSCs from all patients demonstrated a decrease in Pyd in response to MXD (Fig. 3B”). Additionally, changes in transcription of *Col1a1* for COL1 production, *Acta2* for myofibroblast differentiation, and *Plod2* for expression of LH2b attributable to MXD treatment were evaluated (Fig. 4). Compared to NT, significant increases in *Col1a1* (*p* = 0.0077), *Acta2* (*p* = 0.0350), and *Plod2* (*p* = 0.0017) were observed in FSCs stimulated with exogenous TGFβ1, but no significant changes were measured for transcription of *Col1a1* and *Acta2* after administration of MXD in the absence or presence of TGFβ1. In contrast, *Plod2* expression was significantly decreased after MXD treatment in unstimulated (*p* = 0.0084) or TGFβ1-stimulated (*p* = 0.0169) FSCs, in agreement with corresponding decreases in downstream concentrations of Pyd.

## Discussion

Most KOA patients present synoviopathy at various stages of hyperplasia, inflammation, and fibrosis. Because aggressive synovectomy is not part of the standard TKA protocol, most of the diseased synovium resulting from pre-operative KOA and surgical trauma increases the risk for ensuing contraction of the joint capsule [17] due to chronic SFb turnover. Together with fibro-sensitive soft tissue structures that support joint function and stability, SFb limits post-TKA recovery of full ROM. Therefore, evaluation of local and safe administration of targeted pharmacotherapeutics designed to weaken fibrous COL1 deposits in the synovium and neighboring fibro-sensitive structures such as the peri-articular musculature is critical to improve on the success of physical therapy, manual or device-assisted MUA, and arthroscopic lysis of adhesions. As an FDA-approved pharmacotherapeutic, elucidating the antifibrotic potential of MXD to modulate Pyd cross-linking in COL1 fibrous networks in fibroproliferative diseases such as idiopathic pulmonary fibrosis, renal disease, dermal scarring, and metastatic neoplasms in vitro needs further evaluation within the musculoskeletal arena beyond its shown effect on clubfoot fibroblasts [13, 21, 22, 31, 33].

Although a significant decrease in COL1 was measured in unstimulated HFLS and KOA FSCs after treatment with MXD in agreement with studies on clubfoot fibroblasts [21], the effect of MXD was attenuated in the presence of TGFβ1. However, *Plod2*-mediated Pyd formation significantly and consistently decreased with MXD in both the presence and absence of TGFβ1, which warrants further evaluation on a larger sample size. To that end, this work is the first to investigate the effect of MXD on FSCs derived from KOA patients grouped by high and low SFb severity and under TGFβ1 stimulation relative to transcription of *Plod2* and resulting LH2b-mediated Pyd cross-linking. The evidence suggests that MXD can potentially weaken the structural integrity of newly synthesized COL1 by reducing *Plod2* in KOA FSCs to effectively minimize Pyd formation, even in the presence of ongoing stimulation with an average high concentration of TGFβ1 that can be measured from the synovial fluid of KOA patients [44].

High LH2b expression has been measured in the fibrotic synovium of mice with collagenase-induced KOA along with a significant increase in *Plod2* mRNA in a TGFβ1-dependent fashion [34]. Moreover, elevated histological LH2b in this model was associated with high concentrations of lysyl-Pyd and hydroxylysyl-Pyd measured by high performance liquid chromatography [4]. In agreement, upregulated *Plod2* with high expression of LHs has been measured in the human KOA synovium [35], which this study confirms. However, grouping KOA patients by SFb severity strengthens that high LH2b must be specifically required for severe fibrous COL1 deposition (Fig. 1) with Pyd cross-linking while also suggesting that LH2b can potentially inform on SFb status and pose as a direct target for local modulation of SFb [34]. Notably, COL1 and Pyd output measures in association with histological SFb severity status indicate that FSCs at first passage retain a correspondingly fibrotic phenotyp*e*, which allows this approach to become a platform for testing the efficacy of novel antifibrotics to compromise SFb.

To simulate some of the local fibrogenic milieu in vivo, evaluation of antifibrotics on KOA FSCs in vitro must incorporate chronic stimulation with high concentrations of fibrogenic drivers such as TGFβ1, which is elevated in diseased synovial tissues and fluid [5, 30, 44]. In addition to *Col1a1* transcription and myofibroblast differentiation, TGFβ1, through its type 1 kinase receptor, is largely responsible for the upkeep of *Plod*2 activation in fibrotic fibroblasts during idiopathic pulmonary fibrosis, keloid formation, interstitial kidney disease, cirrhosis, and scleroderma [11, 18, 23, 36, 43] to encode for the various LHs that catalyze Pyd [46]. Shao et al. determined that MXD lowers hydroxylysyl and lysyl-Pyd in vitro by directly interfering with TGFβ1 upstream from activation of *Plod2* [41]. However, our work shows that MXD can also have a negative effect on Pyd while FSCs are supplemented with TGFβ1 without significant alteration to transcription of *Col1a1* and *Acta2*. Evaluating the effect of MXD on newly synthesized COL1 from naïve HFLS cells in the presence and absence of TGFβ1 (Fig. 2) allowed for several observations on similar treatment of KOA FSCs. First, untreated, and unstimulated HFLS produced almost 50% less intracellular COL1 than KOA patient FSCs under the same conditions. In addition to increasing the incidence of SMA-positive HFLS compared to unstimulated controls after adjusting for cell number, 4 ng/mL of TGFβ1 [44] adequately stimulated an increase in COL1 production by HFLS to levels produced by FSCs from high SFb sources (Figs. 2D versus 3A). Finally, administration of MXD to either unstimulated HFLS or TGFβ1-stimulated HFLS effectively reduced COL1 by over one third, which suggests that on HFLS, 0.5 µM MXD application overcomes the pro-fibrotic effect of exposure to high levels of TGFβ1.

Unlike in HFLS, MXD did not significantly (Fig. 3A) or consistently (Fig. 3A”) lower COL1 production in KOA FSCs supplemented with TGFβ1. On the other hand, Pyd concentration was significantly (Fig. 3B) and consistently decreased under all conditions and regardless of SFb severity status (Fig. 3B and B”). These data indicate that KOA FSCs primed in vivo for fibrogenesis and exposed to continued TGFβ1 stimulation in vitro respond to MXD by mainly limiting Pyd, which suggests that lower COL1 measured under these conditions could be ruled as a post-translational dysregulation of LH-mediated, COL1 fibril cross-linking rather than a limitation in the ability of KOA FSCs to transcribe *Col1a1*. This is important, because direct inhibition of lysyl-oxidase-like enzymes, which also catalyze Pyd, disrupts the mechano-homeostasis of aberrant scarring in the bronchioalveolar parenchyma engineered by lung myofibroblasts. This results in normalization of COL1 fiber structure in models of idiopathic pulmonary fibrosis, [19] similar to how cross-linking in fibrous COL1 engineered by articular myofibroblasts and fibrotic FSCs could be potentially compromised to increase the effectiveness of peri-operative physical therapy to breakdown arthrofibrosis, normalize soft tissue structure, and restore active ROM.

Expression of *Acta2* signals the presence of SMA-positive stress fibers in myofibroblastic KOA FSCs, which upregulate *Col1a1* and *Plod2* in a TGFβ1-dependent manner (Fig. 4) [1, 32, 34, 35]. The lack of *Acta2* downregulation by MXD in either unstimulated or TGFβ1-stimulated KOA FSCs suggests that MXD did not impair TGFβ1-dependent expression of *Acta2*, which may also help explain why *Col1a1* did not change significantly between MXD-treated compared to NT cultures (Fig. 4). These data suggest that the MXD does not abrogate canonical TGFβ1 functionality, unless a compensatory mechanism exists for pro-fibrotic molecules such as connective tissue growth factor or interleukin-11 [6, 7, 29, 34] to overcome disruption of TGFβ1 receptor activation or downstream Smad signaling for myofibroblast transition and COL1 synthesis to ensue.

Under all conditions and regardless of SFb status, MXD consistently impaired *Plod2* expression (Fig. 4), which supports that MXD targets *Plod2*-mediated Pyd cross-linking by limiting the availability of LH2b, either by direct biochemical interference with hydroxylation of lysine residues [26] or direct suppression of *Plod2* transcription [12, 13, 34]. Notably, MXD lowered but did not abrogate *Plod2* or Pyd, which are still required to preserve the mechanical integrity of the joint capsule [10] and vascular structures [8, 47]. To that end, strategies to modulate Pyd must be carefully designed and the potential for MXD analogs to directly suppress transcriptional regulators of *Plod2*-mediated LH2b expression needs further investigation concurrent with addressing study limitations. First, the study will require increasing the sample size and adjusting observations for confounders that may influence SFb status or interfere with responsiveness to MXD, such as age, BMI, gender, ROM, and co-morbidities (e.g., diabetes). Since these experiments were conducted in vitro, targeted application of MXD analogs in vivo must be evaluated. A KOA mouse model of destabilization of the medial meniscus [14] can be generated in house for that purpose to understand the efficacy and half-life of local MXD administration on pannus formation under a representative milieu of fibrogenic factors released by inflammatory leukocytes, which may not be fully replicated in culture [42]. However, the use of established co-culture models of osteoarthritis such as those integrating FSCs with effector T-cells, polarized macrophages, or defective osteochondral explants [16, 20, 49] could also provide a better understanding of the effects of MXD and novel anti-fibrotics by closely simulating the inflammatory microenvironment in vivo.

Compromising aberrant COL1 deposits through local antifibrotic pharmacotherapy in KOA patients could enhance the success of standard interventions for breaking down scar and maximizing ROM peri-operatively [27] while potentially reducing arthralgia [3]. Repurposing MXD for minimally invasive intra-articular delivery as a prophylactic or minimize progression of severe SFb turnover is especially appealing. This study shows that MXD can limit Pyd cross-linking in KOA patient FSCs, which could weaken new and ongoing turnover of fibrous COL1 deposits without abrogating the fundamental functions of the TGFβ1 cascade and downstream factors related to *Col1a1* transcription. While current interventions for fibrous arthropathy around TKA are not fully guaranteed, intra-articular supplementation with antifibrotics such as MXD analogs or novel small compounds is a promising enhancement strategy that warrants further study.

## Abbreviations

ANOVA: Analysis of variance
APC: Allophycocyanin
BCA: Bicinchoninic acid
BMI: Body mass index
Cat: Catalog
CD: Cluster differentiation
COL1: COL1 type I
Ct: Cycle threshold
DMEM: Dulbecco’s modified eagle medium
DMSO: Dimethyl sulfoxide
DNA: Deoxyribonucleic acid
ELISA: Enzyme-linked immunosorbent assay
FACS: Fluorescence activated cell sorting
FBS: Fetal bovine serum
FDA: Food and Drug Administration
FSCs: Fibroblastic synovial cells
HFLS: Human fibroblast-like synoviocytes
ICC: Immunocytochemistry
IQR: Interquartile range
IRB: Institutional review board
KOA: Knee osteoarthritis
LH: Lysyl hydroxylase
MXD: Minoxidil
NT: Non-treated
PBS: Phosphate-buffered saline
PE: Phycoerythrin
PLOD: Procollagen-lysine,2-oxoglutarate 5-dioxygenase 1
PS: Picrosirius red
qPCR: Quantitative polymerase chain reaction
RNA: Ribonucleic acid
ROM: Range of motion
RT: Room temperature
SEM: Standard error of the means
SFb: Synovial fibrosis
SGM: Synoviocyte Growth Medium
SMA: α-Smooth muscle actin
TGFβ1: Transforming growth factor β1
TKA: Total knee arthroplasty
US: Unstimulated
